# Effect of Volume Fraction on Shear Mode Properties of Fe-Co and Fe-Ni Filled Magneto-Rheological Elastomers

**DOI:** 10.3390/polym14142968

**Published:** 2022-07-21

**Authors:** Shayan Tahir, Muhammad Usman, Malik Adeel Umer

**Affiliations:** 1 School of Civil and Environmental Engineering (SCEE), National University of Sciences and Technology (NUST), H-12 Sector, Islamabad 44000, Pakistan; shyntahir@gmail.com; 2 School of Chemical and Materials Engineering (SCME), National University of Sciences and Technology (NUST), H-12 Sector, Islamabad 44000, Pakistan; umer.adeel@scme.nust.edu.pk

**Keywords:** magnetorheological elastomer, iron, cobalt, nickel, high strains, percentage filler content, base isolator, magnetorheological effect

## Abstract

In this research, the synergistic behavior of magnetorheological elastomers containing nickel and cobalt along with iron particles as magnetically polarizable fillers is examined experimentally under dynamic shear loading. Two different types of magnetorheological elastomer were fabricated having equal proportions of iron and nickel in one kind, and iron and cobalt in the other. The concentrations of magnetic particles in each type are varied from 10% to 40% and investigated for several frequencies, displacement amplitude, and magnetic field values. A test assembly with moveable permanent magnets was used to vary magnetic field density. Force displacement hysteresis loops were studied for dynamic response of magnetorheological elastomers (MREs). It was observed that MREs showed a linear behavior at low strains while nonlinearity increased with increasing strain. The percentage filler content and frequency increased the MRE stiffness whereas it decreased with displacement amplitude. The computed maximum magnetorheological (MR) effect was 55.56 percent. While MRE with iron and cobalt gave the highest effective stiffness, MRE with iron and nickel gave the highest MR effect.

## 1. Introduction

Owing to the life and property damage caused by the earthquakes, different techniques such as vibration damping, and vibration isolation are being used for the seismic protection of a structure [1,2,3]. Over the decades, base isolation has been extensively researched and efficiently used for vibration isolation of a structure [4,5,6]. The principle of base isolation is to provide a flexible layer between the superstructure and its foundation, for which horizontally flexible devices are used [7,8]. Base isolation systems decouple the components of the building from direct contact with the horizontal displacements of the ground so that the transmission of vibration to the primary structure is reduced [6,9]. By providing the flexibility of the support the natural period of the structure is increased [4,10]. This elongation of the natural period helps in shifting the fundamental frequency of the structure away from the dominant frequencies of the ground motion [11,12]. However, conventional base isolators can only respond to the designed earthquake vibrations as their properties cannot be altered once installed [13]. To improve the low-frequency performance of base isolators, research and innovation in materials and design have developed nonlinear techniques in which dynamic properties such as stiffness and damping can be changed, providing a degree of adaptability to passive isolators [14,15].

Magnetorheological Elastomers (MREs) are one such smart material, which when subject to an external magnetic field tend to change their mechanical and rheological properties such as stiffness, natural frequency, and damping capacity [16,17,18,19]. The controllability of the mechanical and rheological properties of MRE makes them resilient over a wide range of disturbance frequencies [20,21]. MRE might have a slower response as compared to Magnetorheological Fluids depending on the type of matrix used, but they do offer more manageable rapid and reversible changes in stiffness as well as damping [22,23]. They also have the additional benefits of fewer sedimentation and leakage issues as compared to MR fluids [24,25].

The MR phenomenon was invented back in the late 1940s and because of the intelligent nature and functionality of MR materials, investigations on their material, fabrication, characterization, and dynamic properties are still ongoing [23,26]. Over the last twenty years, the developments in MR elastomers have been increased significantly and a wide range of their engineering applications have emerged [27,28], particularly where the property changing or the adaptive response feature of MREs is desired as they provide rapid response times with low power requirements [29,30]. These applications include vibration isolators, sensing devices, actuators, magnetic pumping systems, variable damping beams, sandwich beams with the requirement of stiffness or modulus tunability, tool vibration mitigation devices and MRE based actuators for valves [4,17,25,31].

The vast applications of MREs in the engineering world make it necessary to obtain thorough information about the magneto-dynamic characterization of MREs [24,28]. The goal is the study of their mechanical properties under dynamic loading in both the presence and absence of a magnetic field with varying strain amplitude and excitation frequency [32]. Several tests that can be performed based on their mode of operation and direction of the magnetic field include uniaxial and biaxial tension-compression tests and shear tests [29,33]. It is preferred to test MREs in a shear mode more specifically for their practical use in vibration absorbers and base isolators [20] as the earthquake forces tend to have a dominant effect in shear mode [34]. Schubert et al. [35] performed tests on MREs for loading conditions of uniaxial compression, uniaxial tension, and pure shear. Gordaninejad et al. [36] studied the behavior of thick MREs under static compression and double lap shear tests. Additionally, Vatandoost et al. [30], Moreno et al. [24], and Agirre-Olabide [27] studied MRE under compression mode and Dargahi et al. [20], Norouzi et al. [37], and Jung et al. [28] performed shear tests on MREs.

The composition of MREs usually contains a non-polarizable solid-state viscous material for a matrix, polarizable filler material for MR effect, and additives to gain certain properties [16,22,38]. MREs are fabricated by mixing the desired percentages of ferromagnetic particles in the elastomeric matrix with or without the additives. They are then cured in the presence of an external magnetic field for anisotropic MRE, having filler particles arranged in columnar chainlike structures [39]. For isotropic MREs, the filler particles are homogeneously distributed in the matrix as no external magnetic field is applied [33,40]. After curing, the particles are fixed in their respective positions in a solid matrix and upon application of an external magnetic field, the particles are polarized, causing them to align themselves in chains; this is known as the MR effect [23,33,41]. This MR effect is attributed to changes in shear modulus (G), Young’s modulus (E), and stiffness (K) of MRE [17,39], which varies with the different parametric characterization of MREs such as type of matrix material, use of additives, type, percentage content, size, shape, and distribution of magnetic particles, and the external stimuli such as amplitude and direction of a magnetic field [9,29].

The carrier matrix for MREs is usually a vulcanized polymeric viscous material such as natural rubbers, vinyl rubbers, thermoplastic elastomers, silicone rubbers, and polyurethane rubbers [8,42]. When choosing a matrix for MREs, softer rubbers such as silicone rubbers are preferred [23,25] because, when subjected to an external magnetic field, soft elastomeric matrixes provide less resistance against particle movement which allows particle distribution to change vastly [24]. In other words, the softer matrixes lead to a greater MR effect. Another advantage of silicone rubber is its resilience towards heat and chemical attacks [25]. Another optional component in MREs fabrication is additives. Additives in MR elastomers are added for several reasons such as improving its mobility, softening the elastomeric matrix, and decreasing the viscosity, damping ratio, and storage modulus [20]. Silicon oil is quite commonly used as an additive in MREs for preventing agglomeration and forming a homogenous filler particle distribution. It also increases the MR effect by helping in forming a softer polymeric matrix [9].

The most influential component in the composition of MREs is ferromagnetic filler particles as they are responsible for the magnetic field induced response [38]. Commonly and frequently used polarizable material in the making of MREs is carbonyl iron particles (CIPs) [39]. The advantage of using iron is its high saturation magnetization and permeability (which represents its ease of magnetization) along with low remnant magnetization, i.e., the remaining magnetic effect after the external magnetic field is removed [20,34,43]. Researchers have considered it quite suitable for MREs and many studies have targeted the effect of changing the particle concentrations [22,41,44], size [17,32,34,45], shape [32,46], and distribution [40,47] on MREs. Having higher percentages of filler content is desirable, as the distances between particles are reduced and they become more sensitive and responsive to the applied magnetic field [48]. The percentages of filler content that are reported to produce positive results range from 27 percent to 40 percent [23,34,49,50]. Apart from iron fillers, only a few limited choices are available for a selection of polarizable particles for MREs and, among them, cobalt and nickel are reported to be the particles that have this potential [51]. Ni and cobalt are ferromagnetic materials which, unlike iron, do not easily oxidize and have corrosion resistance [52,53]. They are also used in MREs for their good electrical properties [43,54]. There is considerable literature highlighting their use in MRE in pure form and in the form of their ferrites [43,54,55,56]. However, usually, MREs with cobalt and nickel have relatively less MR effect so in order to enhance the magnetic properties of MRE the addition of iron particles along with nickel or cobalt particles is proposed in this study. Considering that the permeability of iron is higher than nickel and cobalt [46,52], mixing it with them can deliver a higher MR effect. Moreover, a detailed study of their stiffness and MR effect with various amplitudes, frequencies and flux value needs to be explored along with the optimum filler percentages.

For this study, two hybrid formulations of MREs were prepared: one by adding iron and cobalt together as a filler and the other one having iron and nickel. Equal quantities of cobalt and iron powders were added for Co-Fe MREs and equal quantities of iron and nickel powders were added as a filler for Ni-Fe MREs. To make a better comparison based on the percentage filler in MREs, the varying percentages of filler material ranging from 10% to 40% by volume of MRE were studied. The dynamic behavior of MREs with hybrid filler was characterized along with its effects on stiffness and magnetization. Each elastomer was added with 10% silicone oil to improve particle dispersion and reduce the hardness of the matrix. PSA and SEM material characterization tests were carried out to investigate particle size, shape, and dispersion in the matrix. To investigate the effect of magnetic flux, frequency, and strain amplitude on the hysteresis and stiffness of MREs, a series of dynamic double lap shear tests were performed. Co-Fe MREs are reported together with Ni-Fe MREs to evaluate and compare their dynamic properties.

## 2. Materials

The components used for the fabrication of MREs were silicone rubber, silicone oil, and powders of iron, cobalt, and nickel. Silicone rubber RTV1505, produced by Shenzhen Rongxingda Polymer Material Co. Ltd., was sourced from Shenzhen, China through Alibaba in the form of part A rubber and part B hardener. The values of hardness, viscosity, elongation, and tensile strength were 5 +/− 2 Shore A, 5000 +/− 500 mPas, ≥550% and ≥2.5 Mpa respectively. Silicon oil was produced and provided by Mingcheng Group Ltd., Dongguan, China. It was used as an additive in the MRE. Iron particles of the size of 3–5 μm were procured by Gongyi City Meiqi Industry & Trade Co., Limited, Gongyi, China. Cobalt and nickel powders were also procured from China. The particle sizes chosen for all three fillers were in micrometers. The quantities of filler material according to percentage content were calculated based on their apparent densities. For material characterization, several tests including SEM, EDS and PSA were performed.

### 2.1. Material Characterization

Particle size analysis was performed for all three filler materials, i.e., iron, cobalt and nickel, using the Horiba laser scattering particle size distribution analyzer, LA-920 (Horiba, Tokyo, Japan). This laser diffraction technique works by matching the scattering patterns, i.e., the size of the sphere that scatters like the particle under test is reported. The results gave the mean diameters of iron, cobalt, and nickel to be 4.778, 7.3503, and 12.3966 μm. The findings revealed that iron particles are the smallest while nickel particles are the largest in this study.

**Figure 1:**
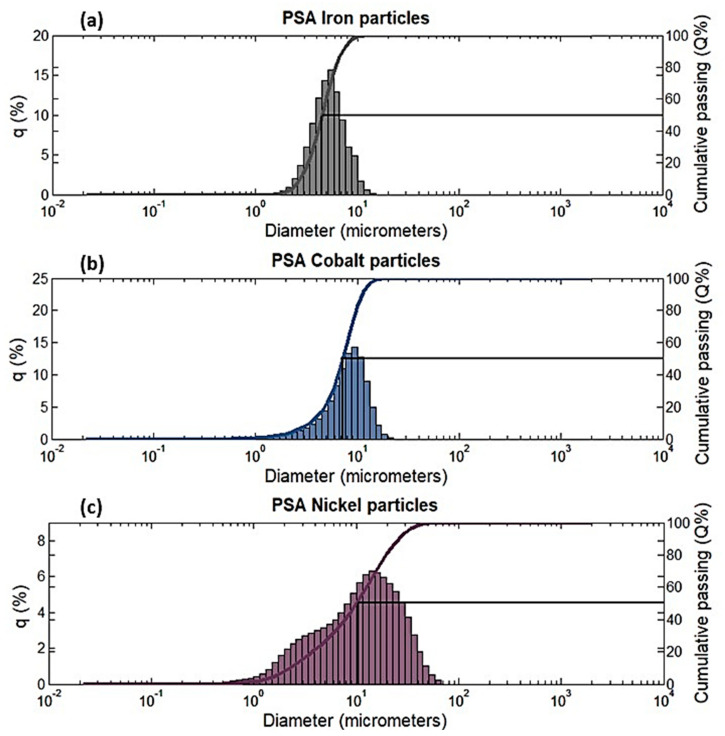
Particle size analysis graphs of (**a**) Iron filler particles (**b**) Cobalt filler particles (**c**) Nickel filler particles.

PSA graphs for all three filler particles are plotted with cumulative passing on the y-axis and particle diameters on the x-axis. The particle size distribution is depicted in Figure 1; the diameter value obtained by cutting the cumulative curve at 50% cumulative passing represents the median. Half of the particles in the distribution have diameters greater than this value, while the other half have diameters less than this value. The median value observed on the graph for iron is between 4 and 5 μm, cobalt is between 7 and 8 μm, and nickel is about 10 μm. The exact values are 4.52, 7.25, and 10.06 μm for iron, cobalt, and nickel respectively.

The highest peak of the histogram represents the mode of distribution, it is the bin with the largest population of particles. In simple terms, most of the particles in the sample were of this diameter. Mode values for iron, cobalt, and nickel are 4.75, 8.21, and 12.39 respectively.

**Figure 2:**
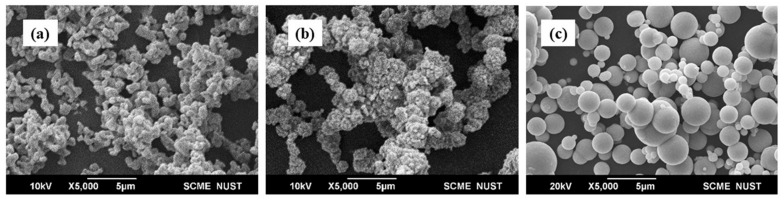
SEM images of (**a**) Cobalt (**b**) Nickel and (**c**) Iron particles.

Figure 2 shows the scanning electron microscope (JEOL JSM-6490A, JEOL, Akishima, Japan) images of cobalt and nickel particles. The size and morphology of the particles are compared at 5 μm and 5000 magnifications. Cobalt particles can be seen to have a more defined shape than nickel particles, but both particles have irregular morphology.

### 2.2. Sample Preparations (Fabrication of MRE)

For the fabrication of MREs, the concentration of each component was calculated along with 15% wastage, as shown in Table 1. To acquire the desired percentage of filler in an MRE, half of the iron particles and the other half of either nickel or cobalt powder were added. The particles were measured using a sensitive weighing balance and were then mixed with part A rubber and 10% silicone oil. Using a bath sonicator of model DSA150-SK2, size: 5.7 l, 920 (Fuzhou Lucky Star Co., Ltd., Fuzhou, Fujian, China) the mixture was sonicated for homogeneous mixing and removal of air bubbles. After half an hour of sonication with intervals of hand mixing the part B rubber was added as a hardening catalyst. Rubber parts A and B were used in a one-to-one ratio. The mixture was then poured into the molds and allowed to cure for 24 h at room temperature. Following this process, 8 pairs of MRE samples with dimensions of 23.62 mm × 12.5 mm × 14 mm were fabricated with filler percentages of 10, 20, 30, and 40%. Four of these pairings had the aforementioned concentrations of iron and cobalt as polarizable fillers, while the other four had varying quantities of iron and nickel. For instance, a 40% Co-Fe MRE was created by adding 40% filler content, which was 20% iron and 20% cobalt. If total filler content in an Ni-Fe MRE is 20% then it holds 10% iron and 10% nickel by volume of MRE.

The composition of the filler mainly depended upon increasing the MR effect of MREs with nickel or cobalt fillers along with less oxidization, corrosion resistance and good electrical properties. The choice of content of filler in the literature was seen to be 10% by volume for MREs with cobalt [51] and was up to 40% considering a critical volume fraction for MREs with iron as a filler [49,50]; so, the whole range from 10 to 40% volume of MRE was tested. The content of filler added is percentage by volume of MRE and adding more than 40% was causing agglomeration of particles, which affected the mixing and dispersion of particles and caused difficulty in curing and bond formation between rubber and filler particles.

**Figure 3:**
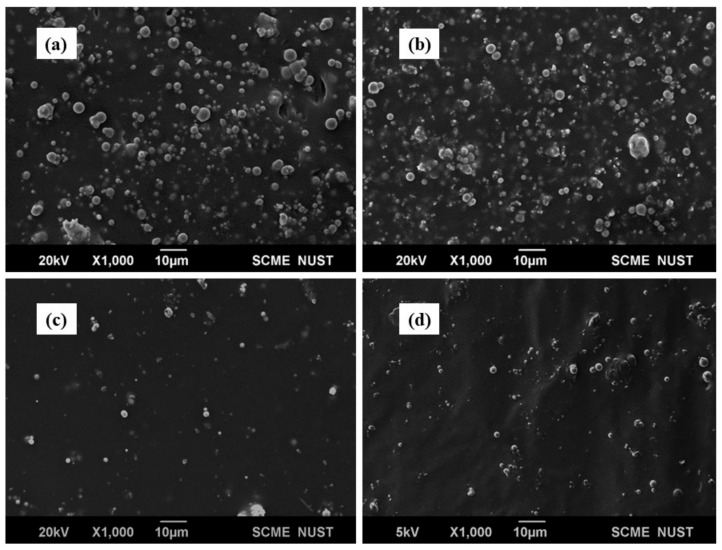
SEM images for MR Elastomers (**a**) 40% Ni-Fe (**b**) 40% Co-Fe (**c**) 10% Ni-Fe (**d**) 10% Co-Fe.

Figure 3 represents the SEM images of the MR Elastomers; the overall dispersion of filler particles in the matrix can be seen.

**Figure 4:**
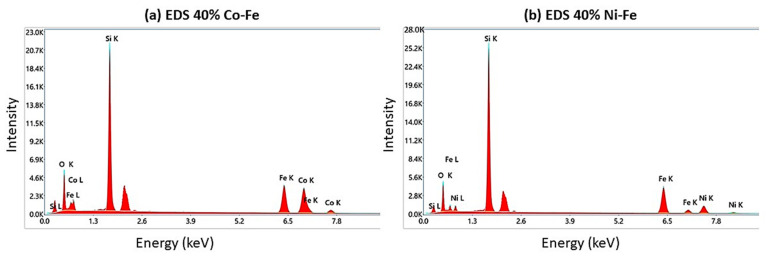
EDS graphs for (**a**) 40% Co-Fe MRE and (**b**) 40% Ni-Fe MRE samples.

To characterize the composition of elements in the elastomer, EDS was performed (Figure 4). The figure represents energy in KeV (kiloelectron volts) on the X-axis and the peak intensity on the Y-axis. The spot analysis was used to verify the presence of silicone, iron, cobalt, and nickel in the elastomeric mix.

## 3. Experiment Setup

**Figure 5:**
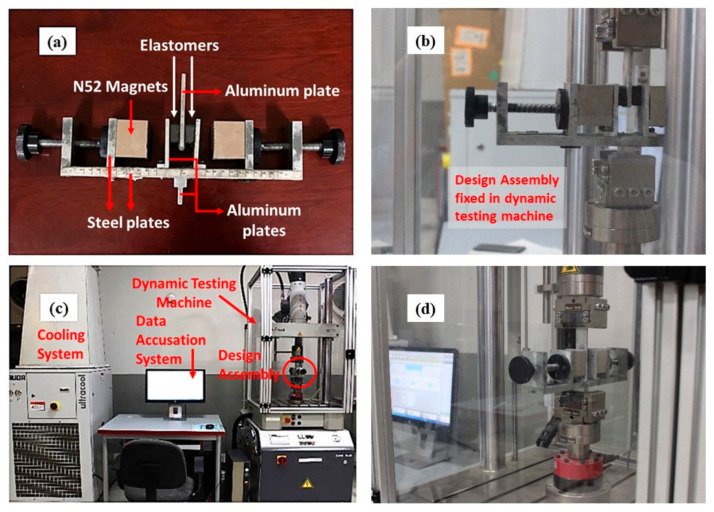
Experimental assembly and set up for dynamic testing (**a**) magnetic assembly holding magnets and elastomers (**b**), (**d**) experimental assembly fixated in the dynamic testing machine (**c**) Zwick/Roell Servohydraulic testing machine used for dynamic loading.

Dynamic shear testing was performed for the mechanical properties’ characterization. The same assembly was used for this study as used by Khayam et al. [34] in their research work. The assembly (Figure 5a) consisted of two grade N52 Neodymium magnets of size 50 mm × 50 mm × 40 mm that could move forward and backward on the mild steel frame, based on the extent of the magnetic field needed. The elastomers were placed between the nonmagnetic aluminum strips with the help of superglue, the upper end of the common middle strip was attached to the machine which moves in shear during testing. The assembly was designed to ensure fairly uniform flux line distribution A specific distance between magnets represents a specific avg flux value calculated using a Gauss meter and FEMM model [34]. The assembly can provide a magnetic field of up to 0.4 Tesla. Additionally, the nonmagnetic aluminum strip helps with concentrating the magnetic lines within the MRE sample.

Dynamic shear tests on MR Elastomers were performed using the Zwick/Roell Servohydraulic testing machine (HC 25) at Zwick lab in National Textile University Faisalabad, Pakistan (Figure 5b–d). The test design is given in Table 2. The tests were performed for frequency values ranging from 0.5 Hz, 1 Hz, 2 Hz, and 3 Hz against displacement values of 4.2 mm, 7 mm, 9.8 mm for 30, 50, and 70% strain. The magnetic field density was varied between values of 0 T, 0.1 T, 0.2 T, 0.3 T, and 0.4 T. The test on every sample was performed for each of the frequency, amplitude, and magnetic flux values. The tests with 5 Hz frequency were also performed against all magnetic flux values but only 4.2 mm amplitude as the machine cannot work for high frequencies and high amplitudes simultaneously. The frequency and amplitude values are provided in the machine while magnetic flux values are changed manually by rotating the knob on the steel strips holding the magnets. The flux values were calculated/confirmed using the Gauss meter and were marked on the scale strip pasted on the frame below. To exterminate the Mullins’s effect, few cycles from the start and end of the test were eliminated from the calculation. The data for each test were acquired via the data acquisition system and were stored within the system.

## 4. Results and Discussions

The data from the double lab shear test were utilized to investigate the dynamic characterization of MREs as well as the effect of different parameters on MRE behavior. Analyzing the obtained data revealed a considerable reliance of the hysteresis and dynamic properties on changing frequency, strain amplitude, and magnetic field density. Each of these impacts is described in greater depth in the following sections. The MREs’ hysteresis and viscoelastic behavior were linked by force-displacement characterization based on experimentally collected data. The shape and slope of the hysteresis loop revealed a lot about the stiffness, damping, energy dissipation, and peak force. All these characteristics were influenced by the changes in the displacement amplitude, strain rate, and magnetic flux.

### 4.1. Effect of Changing Amplitudes

**Figure 6:**
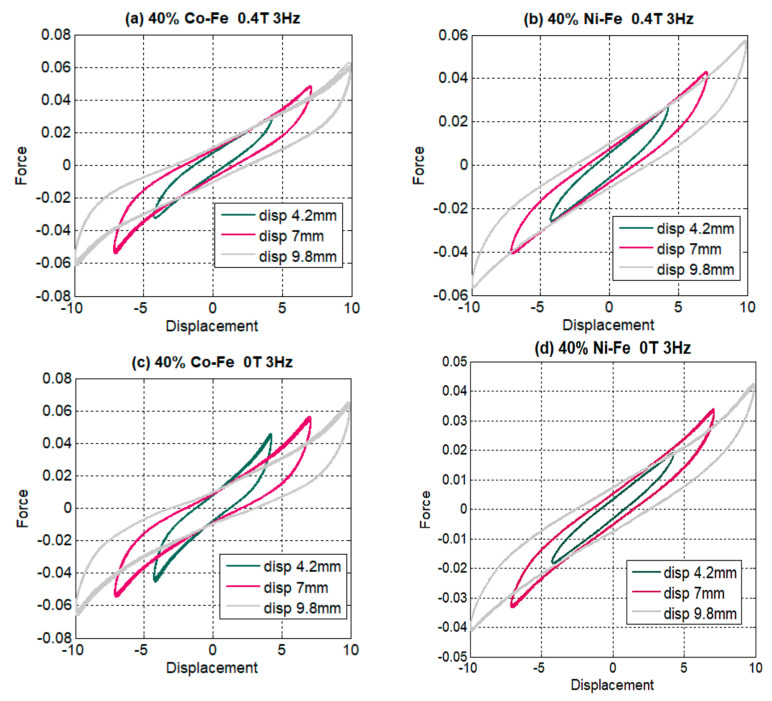
Comparison of amplitude effect on 40 % filler at maximum and minimum flux (**a**) 40% Co-Fe at 0.4 T (**b**) 40% Ni-Fe at 0.4 T (**c**) 40% Co-Fe at 0 T (**d**) 40% Ni-Fe at 0 T.

Figure 6 shows the force-displacement characteristics for the MRE containing 40% filler particles tested at different strain amplitudes for 3 Hz frequency and 0.4 T magnetic field. The slope of hysteresis loops can be seen decreasing with increasing strain amplitude, this implies that the stiffness of MREs decreases with amplitude increase as equivalent stiffness is interpreted as the slope of the major axis of the hysteresis loop. The literature attributes this decrease in stiffness to Payne’s effect. This strain-softening phenomenon has been frequently observed for particle-filled rubbers as the extensibility of polymer chains are affected when filler particles are present, causing the bond between the rubber matrix and the filler particles to be weakened. This can also be observed by shape changes in the hysteresis loop from almost symmetrical and elliptical at low strains to non-elliptical at high amplitudes. This non-linearity and asymmetry in the force at high amplitudes is explained by the large changes produced in the spacing between the filler particles during the loading and unloading of MREs at high strains. The area of the loop also corresponds to the energy dissipation, which is considered an effect of damping, implying that at high amplitudes the large areas of the hysteresis loop signify more energy dissipation and damping.

Now when we compare the effect of strain amplitude with maximum and minimum magnetic fields in Figure 6b,d, the area of loop increases on the application of a magnetic field, offering an increase in energy dissipation with an increase in magnetic field density. The force-displacement graphs also characterize the increase in force with an increase in strain amplitude irrespective of the strain rate (frequency) and magnetic field density. The increased nonlinearity and asymmetry seen in Figure 6c could be because, on the application of a magnetic field, particles interact and arrange themselves but in the absence of a magnetic field there is less interaction between the particles, and when strain is applied it creates more spaces between them, resulting in increased nonlinearity.

**Figure 7:**
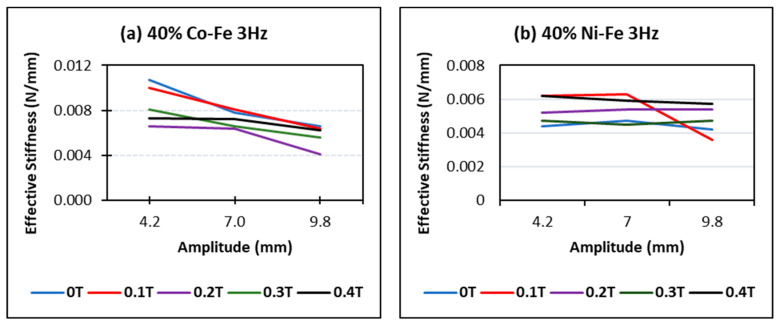
Effect of changing amplitude and flux at maximum filler and maximum frequency on the stiffness of (**a**) Co-Fe MRE (**b**) Ni-Fe MRE.

For effective stiffness and amplitude graphs, the trend of mostly decreasing stiffness with amplitude can be seen (Figure 7); this trend intensifies for high filer percentages, high frequencies, and increased magnetic field. The effective stiffness is calculated using the following Equation (1)

(1) $$k_{eff}=\frac{f_{max}-f_{min}}{d_{max}-d_{min}}.$$

where $f_{max}$ is the maximum force in a data test, $f_{min}$ is the minimum force, $d_{max}$ is the maximum displacement and $d_{min}$ is the minimum displacement.

**Figure 8:**
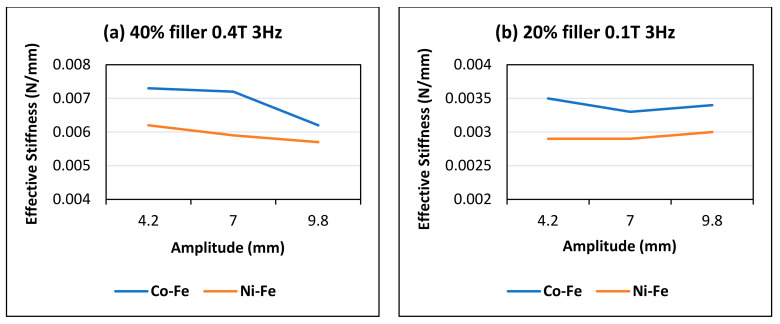
Comparison of amplitude and effective stiffness trends at (**a**) 40% filler and 0.4 T flux (**b**) 20% filler and 0.1 T.

However, in some cases where the values of these parameters are low, the constant stiffness can be seen and for some rare cases, it also tends to increase. This could be because of specific interactions between particles that do not break at high amplitudes. As in Figure 8a, for a sample of higher filler content, the value of stiffness decreases with the amp at high frequency and high magnetic field going from 0.0073 to 0.0062 in the case of Co-Fe and from 0.0062 to 0.0057 in the case of Ni-Fe. In Figure 8b, however, with the case of the sample with low filler content and comparatively low magnetic field, the value of stiffness goes from 0.0035 to 0.0034 in the case of Co-Fe and 0.0029 to 0.003 for the Ni-Fe sample.

### 4.2. Effect of Changing Frequency

**Figure 9:**
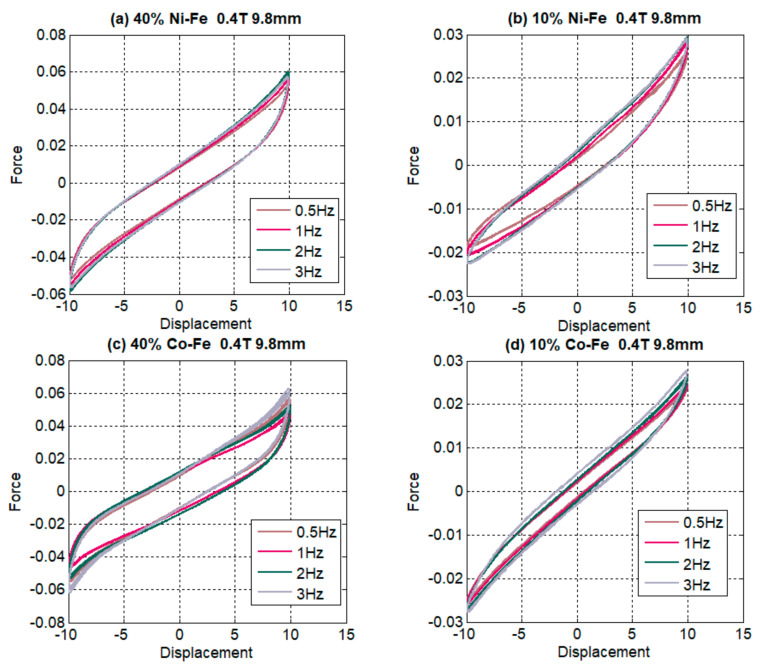
Comparison of force-displacement graphs for changing the frequency at maximum and minimum filler content for Ni-Fe MRE in (**a**,**b**) and Co-Fe MRE in (**c**,**d**).

In Figure 9 trends for different frequencies are plotted in the form of force-displacement hysteresis loops. The comparison of Ni-Fe MREs with Co-Fe MREs is made, representing the filler percentages of 40% and 10% under similar test parameters of a 0.4 Tesla magnetic field and a 9.8 mm displacement amplitude. The peak force is mostly observed at maximum frequency; the trend for force also increases with increasing frequency is seen, although at higher frequencies the effect is less prominent. The wider area of the hysteresis loop indicates an increase in energy dissipation and damping at high frequencies. The steeper slopes of the hysteresis loop indicate the stiffness increase. Furthermore, the graph for effective stiffness also gives a trend of increasing stiffness with increasing frequency. In the literature, this trend is suggested to be a strain rate stiffening effect. 

**Figure 10:**
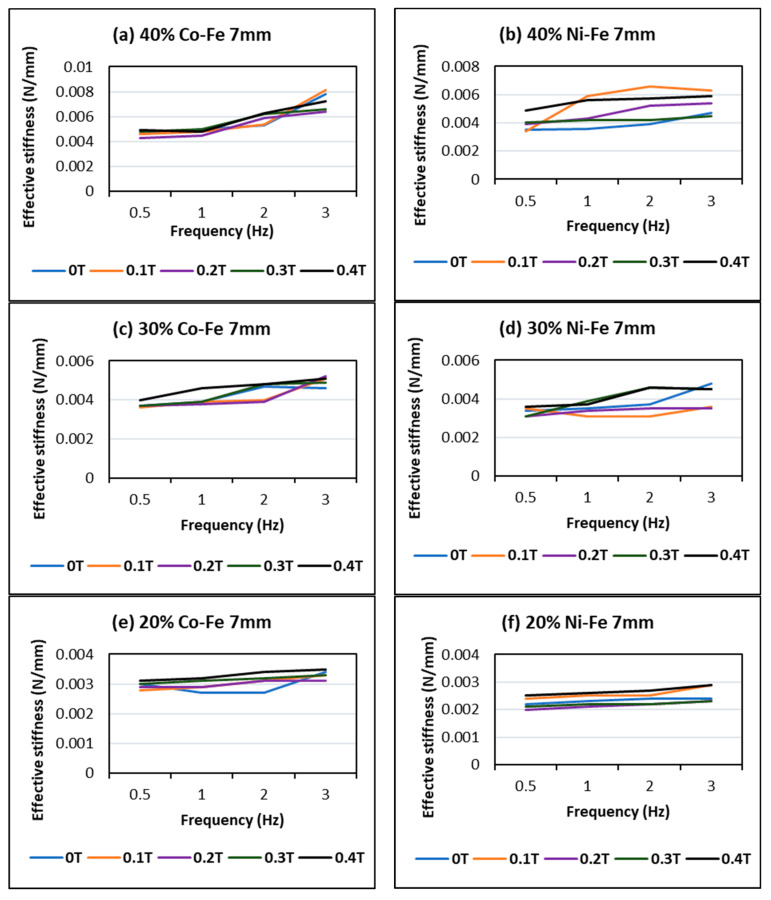

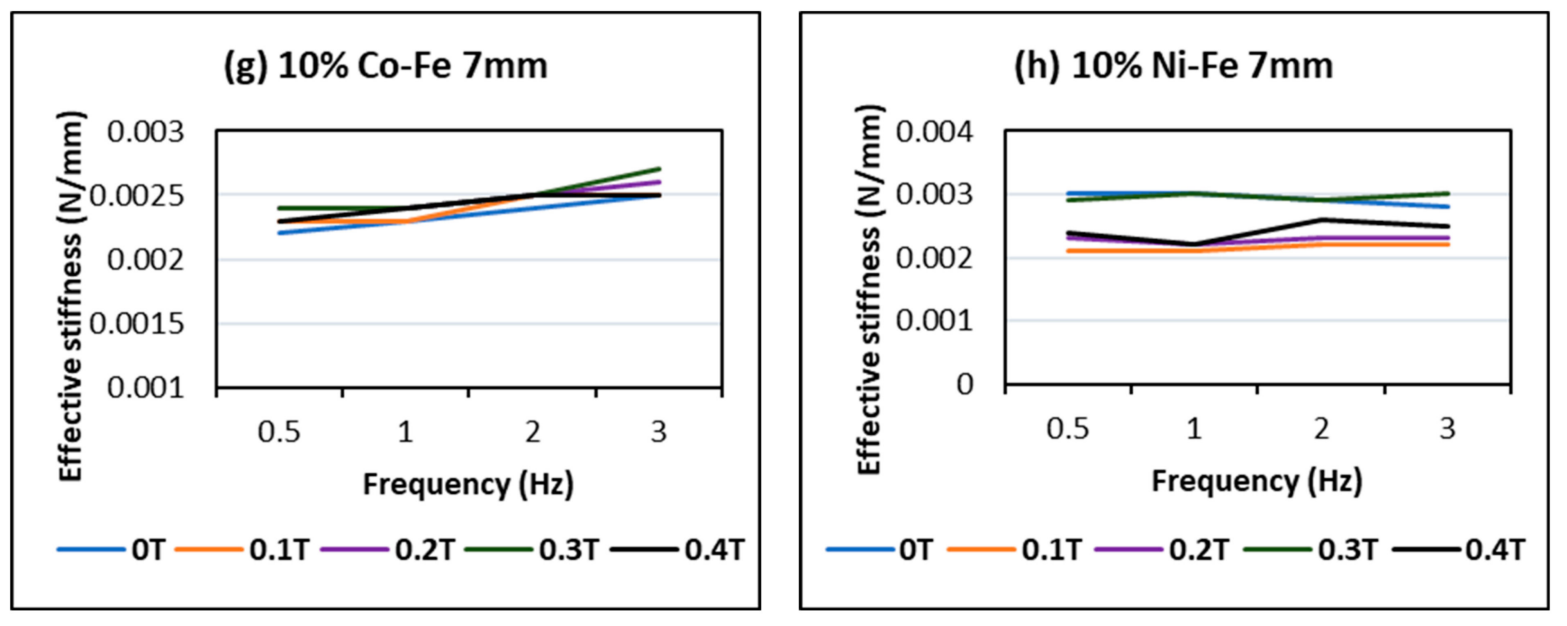
Comparison of % filler content (10, 20, 30 & 40%) between MRE containing Cobalt and Iron (**a**,**c**,**e**,**g**) and MRE having Nickel and Iron (**b**,**d**,**f**,**h**) while simultaneously studying the effect of frequency and magnetic field on effective stiffness.

Figure 10 represents the effect of filler percentage on stiffness increase concerning frequency and magnetic flux at a constant strain amplitude of 7 mm. The trend of gradual increase in the stiffness values can be seen going towards the higher filler percentages, as the larger percentages of filler particles contribute towards a greater confinement of the matrix delivering more non-linearity and stiffness. The MREs containing cobalt and iron have higher stiffness values as compared to the MREs with nickel and iron under similar conditions. For example, in Figure 10d,f when a nickel sample gives the values of peak stiffness of 0.0046 and 0.0029; the cobalt sample in Figure 10c,e for the same test conditions gives values of 0.0051 and 0.0035 respectively. Additionally, 40% filler content has the highest stiffness values among the graphs in Figure 10, the maximum stiffnesses are 0.0066 for Ni-Fe and 0.0081 for Ni-Co.

This trend of stiffness increase at high frequencies is enhanced with the presence of a magnetic field, as under the influence of a stronger magnetic field attraction between the particles increases causing the matrix confinement to increase profoundly, increasing force and stiffness. As can be seen from Figure 10 the highest peak values of stiffness are obtained for high frequencies in the presence of a high magnetic field; this effect is further discussed in the next section.

**Figure 11:**
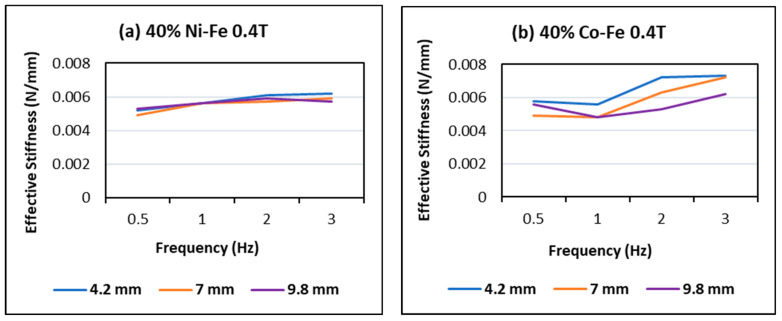
Effect of frequency and amplitude on effective stiffness at 40% filler content and 0.4 T magnetic field on (**a**) Ni-Fe MRE and (**b**) Co-Fe MRE.

The effect of frequency and displacement amplitude on effective stiffness in Figure 11 shows an increase in stiffness with frequency and a decrease with displacement amplitude. Thus, the highest values of stiffness are obtained for high frequency and low amplitude. This trend is more prominent in the case of Co-Fe as compared to Ni-Fe.

### 4.3. Effect of Changing Flux

**Figure 12:**
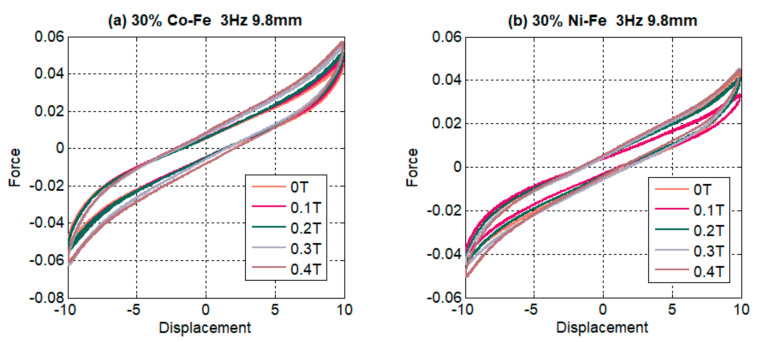
Comparison of (**a**) 30% Co-Fe MRE and (**b**) 30% Ni-Fe MRE force-displacement graphs with respect to changing flux.

The effect of the magnetic field is represented in Figure 12, displaying hysteresis loops for 30% filler content at 3 Hz frequency and 9.8 mm amplitude. The trends are in correspondence with the literature where with the increase in magnetic flux the area of the hysteresis loop grows bigger and wider indicating high energy dissipation and damping [23,30,44]. Force values are also seen to be increasing, going towards the high mag field density and this trend is uniform throughout the samples even though force values for iron-cobalt are higher than those for iron-nickel at the same parameters. The slopes in the force-displacement graph tend to get steeper with the growing magnitude of magnetic flux indicating stiffness increase. 

**Figure 13:**
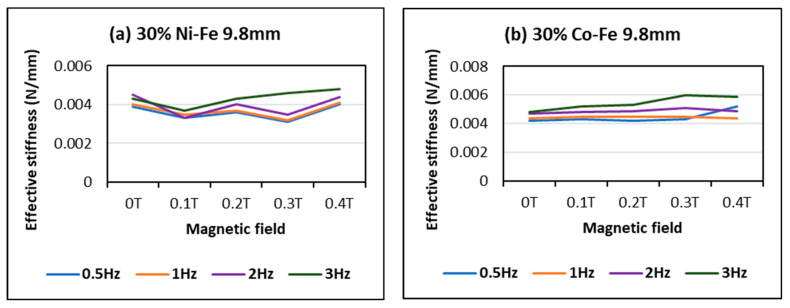
Effect of magnetic field and frequency on effective stiffness of (**a**) 30% Ni-Fe MRE and (**b**) 30% Co-Fe MRE.

The effective stiffness graphs also portray this trend of stiffness increase with flux magnitude and though a few outliners can also be seen. In Figure 13, maximum stiffness values of 0.0048 and 0.0059 can be seen at the highest frequency of 3 Hz and the highest applied magnetic field of 0.4 Tesla for Co-Fe and Ni-Fe samples respectively.

**Figure 14:**
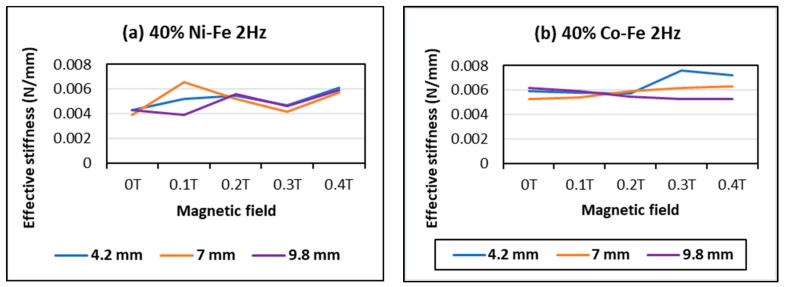
Effect of flux and displacement amplitude on effective stiffness of (**a**) 40% Ni-Fe MRE compared with (**b**) 40% Co-Fe MRE.

Figure 14 represents an evaluation of effective stiffness concerning magnetic field and strain amplitude. The graphs show increasing stiffness for increasing values of magnetic field. As discussed above, the decrease in stiffness with increase in displacement amplitude can also be seen. The comparison of Co-Fe and Ni-Fe MREs shows higher stiffness values for Co-Fe MREs.

**Figure 15:**
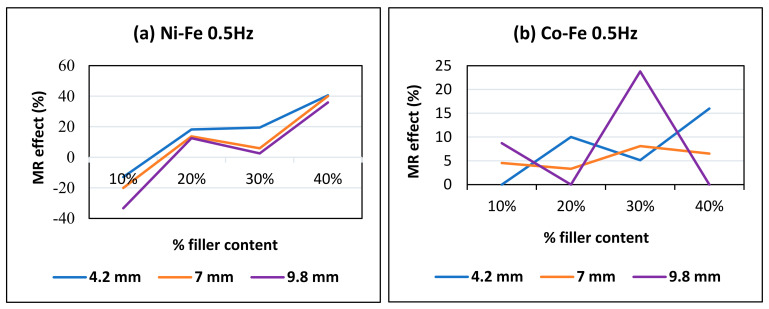
MR effect against every percentage of filler content for (**a**) Ni-Fe MREs and (**b**) Co-Fe MREs.

The MR effect refers to an MRE’s sensitivity to an applied magnetic field [41]. The MR effect (%) here indicates the relative MR effect, which is calculated as the percentage increase in effective stiffness from 0 to 0.4 Tesla using Equation (2). Figure 15 illustrates that the MR effect increases with the percentage of filler particles. This is due to the obvious reason that having a greater percentage of filler particles minimizes the gaps between them, which promotes particle interaction and results in a better response to a magnetic field. The gradual increase with % filler content can be seen in Figure 15a and it is true for all Ni-Fe samples at all frequencies and amplitudes except 30% Ni-Fe. Figure 15b shows a representative MR effect trend for Co-Fe MREs, and this trend of decreasing MR effect for cobalt-iron samples at high % content and high amplitude is consistent for all frequencies. The MR effect is shown to decrease with the large strains, i.e., the MR effect at 9.8 mm is always less than 4.2 mm. The literature explains this deterioration of the MR effect because of the increased spacing between the particles at large shear strains.

(2) $$MR\;effect\;\left(\%\right)=\frac{keff\;\left(0.4T\right)-keff\left(0T\right)}{keff\left(0T\right)}\times 100$$

**Figure 16:**
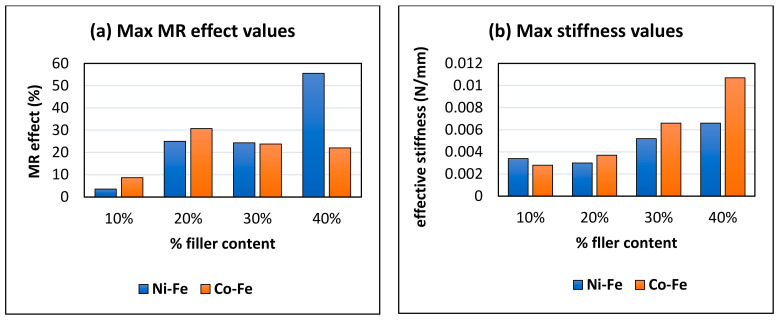
Comparison of (**a**) Maximum MR effect and (**b**) Maximum stiffness values for different %filler contents.

Figure 16a shows the max MR effect values of all eight samples. The maximum value of MR effect is 55.56 at 1 Hz frequency and 7 mm displacement amplitude for 40% Ni-Fe. The MR effect for Ni-Fe samples can be seen increasing with the filler content. The Co-Fe MRE showed a maximum MR effect of 30.76% at 20% filler content, 1 Hz frequency and 4.2 mm displacement amplitude. For Co-Fe samples, increasing the % filler content decreases the MR effect after a certain percentage. So, the optimum percentage for Co-Fe is between 20 and 30%. Figure 16b shows max stiffness values representing Co-Fe having higher stiffness values as compared to Ni-Fe and a maximum stiffness of 0.0107 N/mm.

The max MR effect for Co-Fe MRE is nearly 10% (9.6%) more than what was achieved for isometric cobalt as a polarizable filler in the literature [54]. A study of iron, cobalt, and nickel nanoparticles filled elastomers reported the highest MR effect for iron and the lowest for nickel filled MRE composite [55]. With Ni-Fe MRE an MR effect of 55% is achieved, which is also higher than some iron filled MREs [36]. Other studies with nickel and cobalt filled MREs used different testing modes and thus their MR effect cannot be rightly compared [43,46,56].

## 5. Conclusions

This article studied the effect of displacement amplitude, frequency, and magnetic field on the dynamic behavior of proposed hybrid MREs. The two kinds of MREs fabricated for this study incorporated nickel and cobalt particles along with iron particles as magnetically polarizable fillers. After the careful examination of the results from experimental testing the following conclusions can be obtained:

(1) The MREs with Co-Fe as filler material have a larger Payne effect, i.e., the incremental decrease in effective stiffness of cobalt and iron filled MREs is more than nickel and iron MREs. This trend of strain-softening is intensified in the case of higher frequencies and larger magnetic fields;
(2) The strain rate stiffening effect for both Ni-Fe and Co-Fe was observed to be larger at small strains. For applied magnetic fields, the incremental stiffness increase for Ni-Fe was more than Co-Fe MREs;
(3) The highest MR effect obtained was 55.56% for nickel and iron filled MRE and 30.76% for Co-Fe MRE. The MREs with nickel and iron particles showed a trend of increasing MR effect with increasing % filler content; while for Co-Fe MRE the MR effect increased up to an optimum % and then decreased;
(4) The MREs with cobalt and iron particles produced higher stiffness while the MREs with nickel and iron produced a higher MR effect.

The proposed MREs can be used for gaining better properties than other MREs, for example, instead of adding carbon black or graphene for electrical properties, cobalt and iron could be used, which will provide better rheological properties as well as electrical properties. Other applications include vibration absorbers, sensors, electromagnetic waves absorbers, piezoresistive sensors and dampers.

## Figures and Tables

**Table 1:** Sample ratios and quantities.

Designation	Composition	No. of Samples	% of Particles by Vol of MRE (%)	Filler (g) (Co-Fe or Ni-Fe)	Rubber A = B (mL)	Silicone Oil by 10% Volume of MRE (mL)
40% Co-Fe	Co & Fe	2	20 & 20	5.89–6.08	2.38	0.95
40% Ni-Fe	Ni & Fe	2	20 & 20	3.36–6.08	2.38	0.95
30% Co-Fe	Co & Fe	2	15 & 15	4.42–4.56	2.85	0.95
30% Ni-Fe	Ni & Fe	2	15 & 15	2.52–4.56	2.85	0.95
20% Co-Fe	Co & Fe	2	10 & 10	2.95–3.04	3.33	0.95
20% Ni-Fe	Ni & Fe	2	10 & 10	1.68–3.04	3.33	0.95
10% Co-Fe	Co & Fe	2	5 & 5	1.47–1.52	3.8	0.95
10% Ni-Fe	Ni & Fe	2	5 & 5	0.84–1.52	3.8	0.95

**Table 2:** Test Parameters.

Test Parameters		Cases/Variations
% Filler content	iron + nickel	10%, 20%, 30%,40% (Each having 50% iron and 50% nickel particles)
	iron + cobalt	10%, 20%, 30%, 40% (Each having 50% iron and 50% cobalt particles)
Flux		0 T, 0.1 T, 0.2 T, 0.3 T, 0.4 T
Frequency		0.5 Hz, 1 Hz, 2 Hz, 3 Hz, 5 Hz
Disp. Amp		4.2 mm (30% strain), 7 mm (50% strain), 9.8 mm (70% strain)

## Data Availability

The raw/processed data required to reproduce these findings cannot be shared at this time as the data also forms part of an ongoing study.
